# MiR-1976 knockdown promotes epithelial–mesenchymal transition and cancer stem cell properties inducing triple-negative breast cancer metastasis

**DOI:** 10.1038/s41419-020-2711-x

**Published:** 2020-07-03

**Authors:** Jingyi Wang, Minghui Li, Xu Han, Hui Wang, Xinyang Wang, Ge Ma, Tiansong Xia, Shui Wang

**Affiliations:** 1 Department of Breast Surgery, The First Affiliated Hospital with Nanjing Medical University, 300 Guangzhou Road, Nanjing, 210029 China; 2 Jiangsu Key Lab of Cancer Biomarkers, Prevention and Treatment, Jiangsu Collaborative Innovation Center for Cancer Personalized Medicine, School of Public Health, Nanjing Medical University, Nanjing, 211166 China

**Keywords:** Breast cancer, Metastasis

## Abstract

Triple-negative breast cancer (TNBC), characterized by high aggression and invasiveness, has a worse prognosis than other subtypes of breast cancer. Establishing a novel animal model is helpful to understand the mechanisms involved in the progress of TNBC metastasis. In a self-established mouse model consisting normal human breast tissues and normal human bone tissues, TNBC cell line SUM-1315 could spontaneously form species-specific bone metastasis. The expression level of miR-1976 in SUM-1315-bo (derived from metastatic bone tumor) was found lower than that in SUM-1315-br (derived from orthotopic breast tumor). MiR-1976 was found to be downregulated in TNBC tissues, and lower expression of miR-1976 was correlated with worse overall survival in a patient cohort obtained from TCGA database. MiR-1976 knockdown promoted epithelial–mesenchymal transition (EMT) and cancer stem cell (CSC) properties in vitro and in vivo. Phosphatidylinositol-4,5-bisphosphate 3-kinase catalytic subunit gamma (PIK3CG) was verified as a target gene by sequencing, biotinylated miRNA pull-down, and luciferase reporter assay. Moreover, overexpression and suppression analysis implicated PIK3CG as a mediator of the biological effects of miR-1976. Our study demonstrated that miR-1976 knockdown could promote EMT and CSCs by PIK3CG. These findings may reveal mechanisms of TNBC metastasis, and represent a potential treatment target for patients with TNBC.

## Introduction

Triple-negative breast cancer (TNBC), characterized by high aggression and invasiveness, is an unsolved difficulty in treatment, and metastasis is the major driver of death^1–3^. For better treatment of TNBC metastasis, it is urgent to understand the biological characteristics. The mechanisms of metastasis are complex, including the effects between tumor cells and microenvironment, the formation of circulating tumor cells (CTCs), the interaction with target organs before the implantation of CTCs, the circulating free DNA, and so on^4–7^.

Establishing a novel animal model is helpful to understand the mechanisms of TNBC metastasis^8,9^. Common animal models are unable to fully simulate the process of metastasis, mainly due to the lack of the microenvironment of normal human breast tissues and target organs^10,11^. Based on the NOD/SCID-hu system, our group established a novel mouse model, in which normal human breast and bone tissues were subcutaneously transplanted at different abdomens of a mouse^12^. After testing the biology of several human breast cancer cell lines in the model, we found that TNBC cell line SUM-1315 could spontaneously form species-specific bone metastasis, certifying the model.

MicroRNAs (miRNAs) expression profiling analyses of SUM-1315-br (derived from orthotopic breast tumor) and SUM-1315-bo (derived from metastatic bone tumor) were conducted. MiRNAs are a class of small non-coding RNAs that regulate gene expression^13^. Comparing the differences in miRNAs expression profiling analyses of SUM-1315-br and SUM-1315-bo, key molecules promoting TNBC metastasis could be screened out.

In the study, the expression level of miR-1976 in SUM-1315-bo was found lower than that in SUM-1315-br. The evaluation of clinical TNBC specimens also showed that miR-1976 was downregulated in malignant tissues and lower expression of miR-1976 was associated with worse overall survival in a patient cohort obtained from TCGA database. The biological functions of miR-1976 in TNBC metastasis were investigated in vitro and in vivo, and further identified phosphatidylinositol-4,5-bisphosphate 3-kinase catalytic subunit gamma (PIK3CG) as a direct target gene of miR-1976. MiR-1976 knockdown promoted epithelial–mesenchymal transition (EMT) and cancer stem cell (CSC) properties by targeting PIK3CG in TNBC metastasis. Moreover, miR-1976 decreased the expression of PIK3CG and restoration of PIK3CG expression attenuated the inhibitory effects of miR-1976 on EMT and CSCs in TNBC. Thus, miR-1976 may serve as an anti-cancer metastatic biomarker with high efficacy.

## Methods

### Clinical samples

TNBC tissues and adjacent normal tissues (35 pairs) were obtained from the First Affiliated Hospital with Nanjing Medical University. All patients received no neoadjuvant therapy. The collected samples were frozen in liquid nitrogen immediately after resection. All patients provided written informed consent, and the study was approved by the Ethics Committee of the First Affiliated Hospital with Nanjing Medical University.

### Analysis of TCGA database

The correlation between the expression level of miR-1976 and the overall survival of patients with TNBC was analyzed by Kaplan–Meier Plotter (https://kmplot.com/analysis/). Patients were separated by the auto select best cutoff, which was computed between the lower and upper quartiles and was the best performing threshold.

### Cell lines and culture condition

Primary breast cancer cell lines (SUM-1315-br and SUM-1315-bo) were purified from orthotopic breast tumor and metastatic bone tumor respectively. SUM-1315 was kindly provided by Stephen Ethier (University of Michigan, Ann Arbor, MI, USA). Other human breast cancer cell lines (MDA-MB-231, ZR-75-1 and MCF-7) were obtained from the American Tissue Culture Collection (ATCC). All cell lines were cultured in Dulbecco's modified Eagle medium (DMEM) medium (Gibco, Detroit, MI, USA) containing 10% fetal bovine serum (Gibco, Detroit, MI, USA) and 1% penicillin–streptomycin (Gibco, Detroit, MI, USA) at 37 °C with 5% CO_2_.

### Lentivirus and plasmid transfection

The miR-1976 negative control, mimics and inhibitor lentiviruses were constructed by GenePharma (Shanghai, China) to change the expression level of miR-1976 in breast cancer cell lines. The transfection of lentiviruses was performed according to the respective MOI. Stable cell lines were selected by using 5 μg/ml puromycin (Sigma, USA). Plasmid of target gene PIK3CG was constructed by GenePharma (Shanghai, China). Cells were seeded into 6-well plates 1 day before plasmid transfection, and transfections were performed using Lipofectamine 3000 (Invitrogen, USA) according to the manufacturer’s protocol.

### Cell wound-healing assay

Cell wound-healing assay was used to evaluate cell migration. Cells were seeded in 6-well plates and a monolayer was scratched after incubation for 24 h. After washing three times with PBS, phase images were taken by inversion fluorescence microscopy at 0 and 48 h (Olympus, Japan). ImageJ software was used to measure the wound areas.

### Cellular transwell assay

One-hundred microliters of cell suspension in serum-free DMEM was added to the upper chamber and 600 μl DMEM containing 10% fetal bovine serum was added to the lower chamber to induce cells to the other side of membrane. After incubation for 24 h, cells were collected to assess migration. When assessing invasion, membranes were coated with Matrigel (Corning, USA), and cells were collected after incubation for 48 h. Then cells that passed through the membrane were stained with crystal violet (Beyotime, Shanghai, China) for 30 min, and counted at a ×200 magnification in five randomly selected regions per well.

### Cell viability assay

Cells were seeded at a density of 1 × 10^4^/well in 96-well plates in triplicate and incubated for 24 h. Cell viability was assayed by CellTiter-Lumi™ Luminescent Cell Viability Assay Kit (Beyotime, Shanghai, China) based on quantification of adenosine triphosphate (ATP), which represented the number of active cells. Data were presented as relative viability by comparing with negative control, the viability of which is assumed to be 1.

### Cell apoptosis analysis

Cell apoptosis rates were analyzed after transfection with miR-1976 mimics or inhibitors. Cells were collected after washing twice with PBS, and were stained with the Annexin V-APC/7-AAD apoptosis kit (MULTI SCIENCES, Hangzhou, China) for 5 min at room temperature. They were then evaluated by flow cytometry (BD Biosciences, USA).

### Flow cytometry analysis

The expression levels of Ki-67, CD44 (Cluster of differentiation 44) and CD24 (Cluster of differentiation 24) were analyzed by flow cytometry.

Cells were dissociated using Trypsin Solution without EDTA (Beyotime, Shanghai, China) and resuspended in Flow Cytometry Staining buffer (MULTI SCIENCES, Hangzhou, China). Cells were stained with anti-human Ki-67 (BioLegend, USA) after permeabilization with 70% ethanol at −20 °C for 1 h. Data were compared by the mean of log fluorescence intensity. Cells were incubated with anti-human CD44-APC, anti-human CD24-PE and the APC and PE isotype control antibodies (eBioscience, USA) for 30 min at room temperature. Cells were washed and evaluated by flow cytometry (BD Biosciences, USA) to determinate population distribution.

### Mammosphere formation assay

Cell lines were plated in ultra-low attachment 6-well plates (Corning, USA) at a density of 5000/well in serum-free DMEM/F12 medium supplemented with B27 (Invitrogen), 20 ng/ml EGF (Invitrogen), and 20 ng/ml bFGF (BD Biosciences). After culturing for 7–14 days, mammospheres greater than 60 μm in diameter were counted under the microscope. Experiments were done in triplicates.

### RNA extraction and quantitative real time-polymerase chain reaction (qRT-PCR)

Total RNA was extracted by Trizol reagent (Takara, Japan) following the manufacturer’s protocol. For miRNA expression analysis, complementary DNA (cDNA) was specifically synthesized and miRNA was detected with Bulge-Loop™ miRNA qRT-PCR (Ribobio, Guangzhou, China). Relative expression level of miR-1976 was normalized to U6. For PIK3CG messenger RNA (mRNA) expression analysis, first strand cDNA was synthesized by PrimeScript™ RT Master Mix (Takara, Japan). PIK3CG mRNA expression was detected with TB Green^®^ Premix Ex Taq™ II (Takara, Japan) and normalized to GAPDH. The qRT-PCR reactions were performed in the ABI StepOne Plus (Applied Biosystems, Foster City, CA, USA) and the relative expression was calculated using the 2^–∆∆ct^ method. All procedures were performed in triplicate.

### Western blotting

Protein was extracted from cells, electrophoresed on 10% sodium dodecyl sulfate–polyacrylamide gel electrophoresis and transferred to polyvinylidene fluoride (PVDF) membranes. Then membranes were blocked in QuickBlock™ Blocking Buffer (Beyotime, Shanghai, China) for 15 min, and incubated in primary antibodies for 4 °C overnight. After washing with Tris Buffered saline Tween (TBST), the membranes were incubated in secondary antibodies for 2 h at room temperature. The primary antibodies included anti-E-cadherin, anti-N-cadherin, anti-Vimentin, anti-Snail, anti-Slug, anti-CD44, anti-PIK3CG, anti-AKT, anti-p-AKT and anti-GAPDH (diluted 1:1000, Cell Signaling Technology, USA). GAPDH was used as an internal control.

### Transcriptome sequencing

Total RNA samples of SUM-1315 transfected with negative control and mimics lentiviruses were qualified by agarose gel electrophoresis and quantified by NanoDrop. The commercial kit (DECODE GENOMICS, Nanjing) for mRNA library preparation was used. The differentially expressed mRNAs were analyzed above the threshold level (1.5) and *p*-value was less than or equal to 0.05 between SUM-1315 transfected with negative control and mimics.

### Biotinylated miRNA pull-down

SUM-1315 cell was transfected with the biotinylated negative control or mimics (Ribobio, Guangzhou, China), and digested 24 h after transfection. Before preparing lysate, crosslink was performed. Then mRNA was isolated from crude lysate by incubating with Streptavidin C1 (Invitrogen, USA) following the protocol. Then biotin-miRNA-mRNA were pulldown, and the RNA was extracted directly from the remaining beads with Trizol reagent (Takara, Japan).

### Luciferase reporter assay

Possible binding sites between miR-1976 and 3ʹ-UTR of PIK3CG were obtained from TargetScan. Wild-type PIK3CG plasmid (PIK3CG-3ʹ-UTR-WT) and mutant PIK3CG plasmid (PIK3CG-3ʹ-UTR-MUT) were purchased from Genechem (Shanghai, China). Cells were transfected with appropriate plasmid. Luciferase assays were measured by the Dual-luciferase reporter assay system after transfection 48 h (Promega, USA). Transcriptional activity was calculated as Luciferase activity/Renilla Luciferase activity.

### Animal models

All animal experiments were approved by the Nanjing Medical University Institutional Animal Care and Use Committee and were carried out at the Animal Center. In total, two groups with ten 4-week-old female mice (BALB/c nude mice) per group were used for the construction of a tail vein metastasis model. In all, 1 × 10^6^ cells (SUM-1315 miR-NC or SUM-1315 miR-mimics) were injected into the tail vein of each female nude mouse. All mice were euthanized after 4 weeks, and lungs were removed and fixed with 4% paraformaldehyde. Three sections were stained with hematoxylin-eosin (HE) to assess the presence of metastasis.

### Immunohistochemical analyses (IHC)

Four-micrometer-thick sections were made from the formalin-fixed, paraffin-embedded blocks, which were then dried, deparaffinized, and rehydrated following the manufacturer’s protocol. The anti-E-cadherin, anti-N-cadherin, anti-Vimentin, and anti-CD44 incubation was carried out at room temperature for 1 h after antigen retrieval. The detections were conducted by standard avidin-biotin-peroxidase techniques. Immunohistochemical samples were assessed by two pathologists, and the staining intensity was defined as follows: weak (0); moderate (1); strong (2).

### Statistical analysis

The statistical analysis was performed by SPSS software (Version 25.0) and GraphPad Prism (Version 8.0), and presented as mean ± standard deviation (SD). All data were analyzed by two-tailed Student’s *t*-test. Each experiment was repeated at least three times, and *p* < 0.05 was considered statistically significant.

## Results

### MiR-1976 was significantly downregulated in TNBC and acted as a prognostic factor

**Fig. 1:**
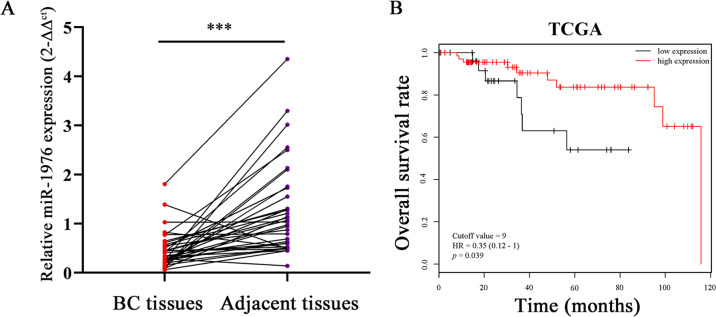
MiR-1976 was significantly downregulated in TNBC and acted as a prognostic factor. **a** The expression levels of miR-1976 in TNBC tissues were lower than those in adjacent normal tissues. **b** Data of miR-1976 were obtained from TCGA database. Lower expression of miR-1976 was correlated with worse overall survival in patients with TNBC. ***p < 0.001.

To identify differentially expressed miRNAs between SUM-1315-br and SUM-1315-bo, miRNA expression profiling analysis was performed. MiR-1976 was found significantly lower in SUM-1315-bo than that in SUM-1315-br, and the result was verified by qRT-PCR (Supplementary Fig. S1a). The expression levels of miR-1976 in TNBC cell lines (SUM-1315 and MDA-MB-231) were lower than those in hormone receptor-positive cell lines (ZR-75-1 and MCF-7) (Supplementary Fig. S1b). The expression levels of miR-1976 in 35 pairs of TNBC tissues and adjacent normal tissues were also examined by qRT-PCR, and the expression levels were markedly decreased in TNBC tissues (Fig. 1a). Clinical characteristics of individuals were shown in Supplementary Table S1. Data on miR-1976 were obtained from 97 patients with TNBC from the TCGA database and analyzed by Kaplan–Meier Plotter. The lower expression of miR-1976 was correlated with worse overall survival (68 high expression and 29 low expression, *p* = 0.039), and the cutoff value was 9 and HR was 0.35 (95% CI 0.12–1) (Fig. 1b).

### miR-1976 knockdown promoted EMT in vitro

**Fig. 2:**
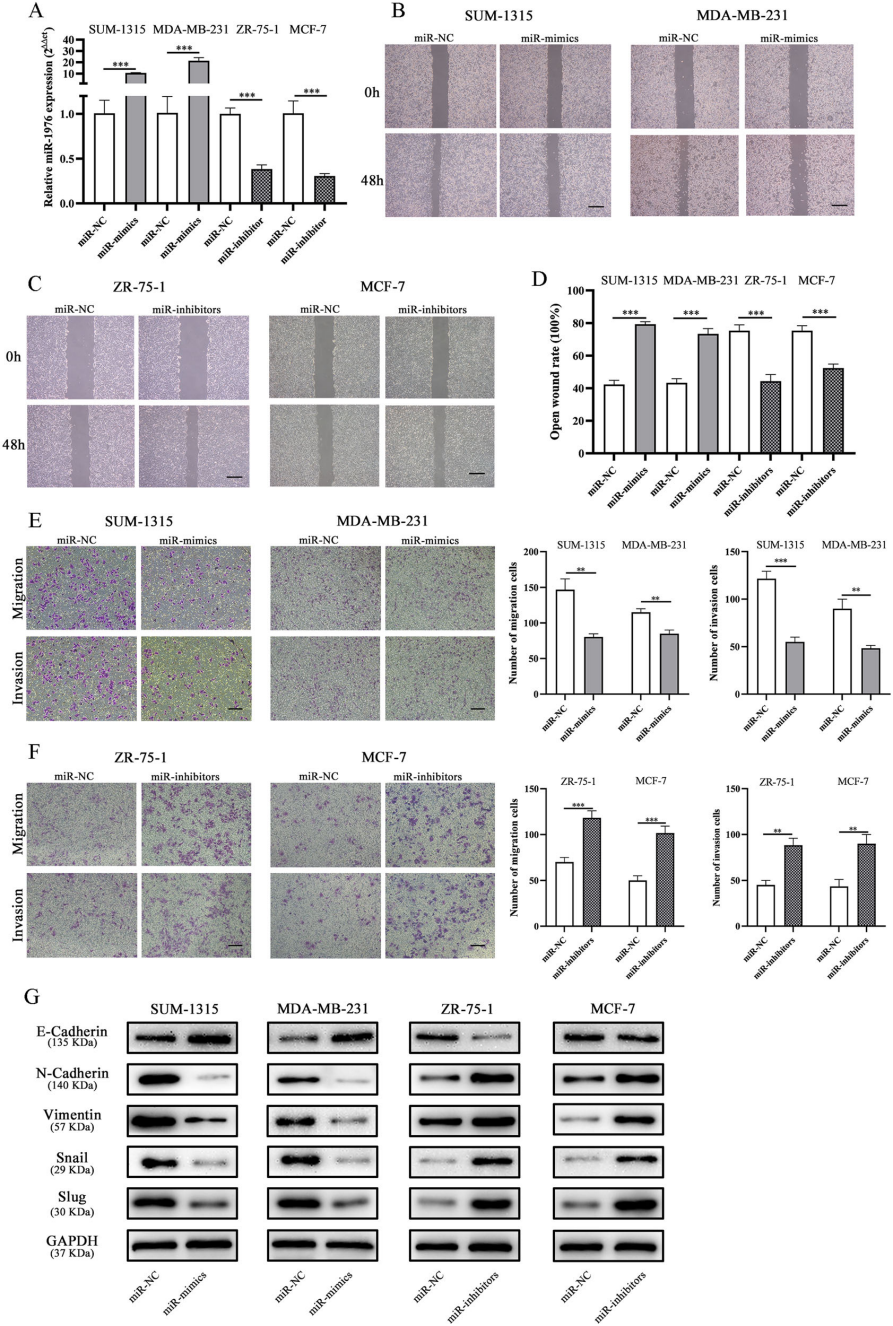
MiR-1976 knockdown promoted migration and invasion in vitro. **a** The expression levels of miR-1976 in breast cancer cells transfected with miR-1976 mimics and inhibitor lentivirus respectively were tested by qRT-PCR. **b**–**d** The wound healing assays were performed to assess the effect of miR-1976 on cell motility at 0 and 48 h. Scale bar: 100 μm. **e**, **f** The transwell migration and invasion assays were performed to detect the effects of miR-1976 on migration and invasion. Scale bar: 100 μm. **g** Western blot analysis showed that miR-1976 mimics increased the level of E-Cadherin, and reduced the levels of N-Cadherin, Vimentin, Snail, and Slug in SUM-1315 and MDA-MB-231, vice versa in ZR-75-1 and MCF-7 transfected with miR-1976 inhibitor. *p < 0.05, **p < 0.01, ***p < 0.001. The data ex*p*ressed as the mean ± SD.

TNBC cell lines (SUM-1315 and MDA-MB-231) were transfected with miR-1976 mimics lentivirus, and hormone receptor-positive cell lines (ZR-75-1 and MCF-7) were transfected with miR-1976 inhibitor lentivirus. The transfection efficiency was verified by qRT-PCR (Fig. 2a). Cell wound-healing assay and cellular transwell assay were used to assess the effects of miR-1976 on cell motility. As shown in Fig. 2b, the wound healing rate of SUM-1315 and MDA-MB-231 transfected with miR-1976 mimics was significantly decreased compared with negative control, while ZR-75-1 and MCF-7 transfected with miR-1976 inhibitor showed the opposite effects (Fig. 2c, d). Furthermore, the results of transwell assays revealed that miR-1976 mimics significantly attenuated the migration of SUM-1315 and MDA-MB-231 compared with negative control, while miR-1976 inhibitor enhanced cell migration (Fig. 2e, f). EMT markers were detected by western blotting. The epithelial marker, E-Cadherin, was obviously upregulated, but mesenchymal markers and transcription factors, N-Cadherin, Vimentin, Snail and Slug, were markedly downregulated in SUM-1315 and MDA-MB-231 cells transfected with miR-1976 mimics, while the effects in ZR-75-1 and MCF-7 transfected with miR-1976 inhibitor were reverse (Fig. 2g).

The effects of miR-1976 on proliferation and cell death were also analyzed by cell viability assay, flow cytometry analysis of Ki-67 and cell apoptosis analysis. MiR-1976 mimics suppressed cell viability, reduced the expression level of Ki-67, and induced apoptosis in SUM-1315 and MDA-MB-231 compared with negative control, and miR-1976 inhibitor in ZR-75-1 and MCF-7 had the opposite effects (Supplementary Fig. S2).

### miR-1976 knockdown enhanced CSC properties

**Fig. 3:**
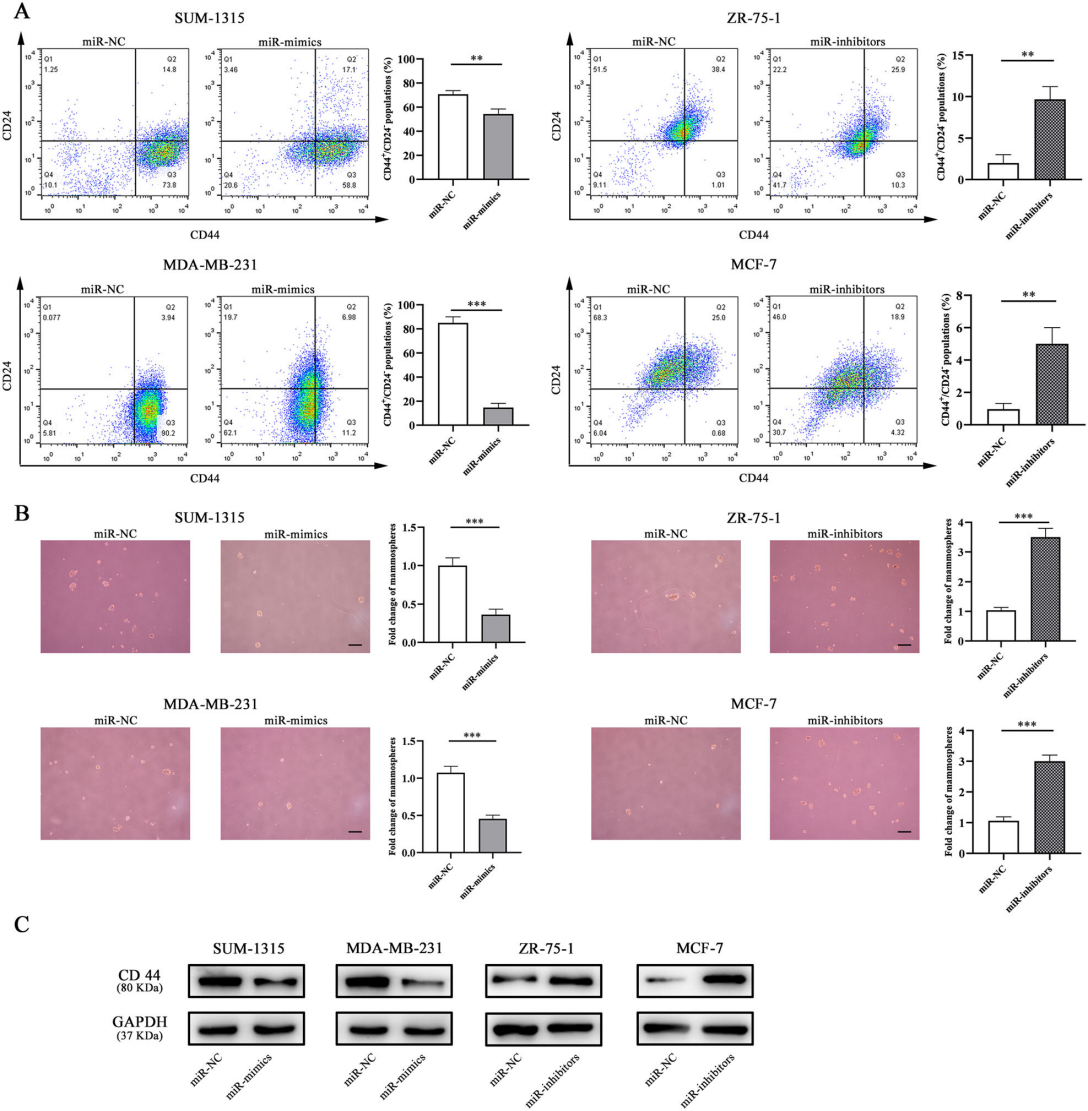
MiR-1976 knockdown changed CSC properties. **a** The expression levels of CD44 and CD24 in cells transfected with miR-1976 mimics and inhibitor respectively were analyzed by flow cytometry analysis. Cells were stained with anti-CD44-APC and anti-CD24-PE. Overexpression of miR-1976 decreased the populations of CD44^+^/CD24^−^ in SUM-1315 and MDA-MB-231, and miR-1976 knockdown increased the populations of CD44^+^/CD24^−^ in ZR-75-1 and MCF-7. **b** Mammosphere formation assay was performed, and the representative pictures were shown. Scale bar: 100 μm. **c** Western blot analysis showed that miR-1976 reduced the protein expression level of CD44 in SUM-1315 and MDA-MB-231, and miR-1976 inhibitors increased the level in ZR-75-1 and MCF-7. *p < 0.05, **p < 0.01, ***p < 0.001. The data expressed as the mean ± SD.

CSC properties were analyzed by flow cytometry analysis and sphere formation assay. As shown in Fig. 3a, miR-1976 mimics reduced the populations of CD44^+^/CD24^–^ in SUM-1315 and MDA-MB-231 cells compared with negative control, and miR-1976 inhibitor increased the populations in ZR-75-1 and MCF-7 cells. Cells stained with the APC and PE isotype control antibodies were shown in Supplementary Fig. S3. Consistent with the results of flow cytometry analyses, miR-1976 mimics decreased the mammosphere formation capacity, and miR-1976 inhibitor increased the capacity (Fig. 3b). Next, western blot analysis was performed to examine CSCs marker CD44. Consistently, the results showed that the protein expression of CD44 was obviously reduced in SUM-1315 and MDA-MB-231 cells transfected with miR-1976 mimics. In contrast, miR-1976 inhibitor increased the protein expression of CD44 (Fig. 3c). Our results indicated that miR-1976 knockdown enhanced the CSCs.

### Overexpression of miR-1976 reduced tumor metastasis in vivo

**Fig. 4:**
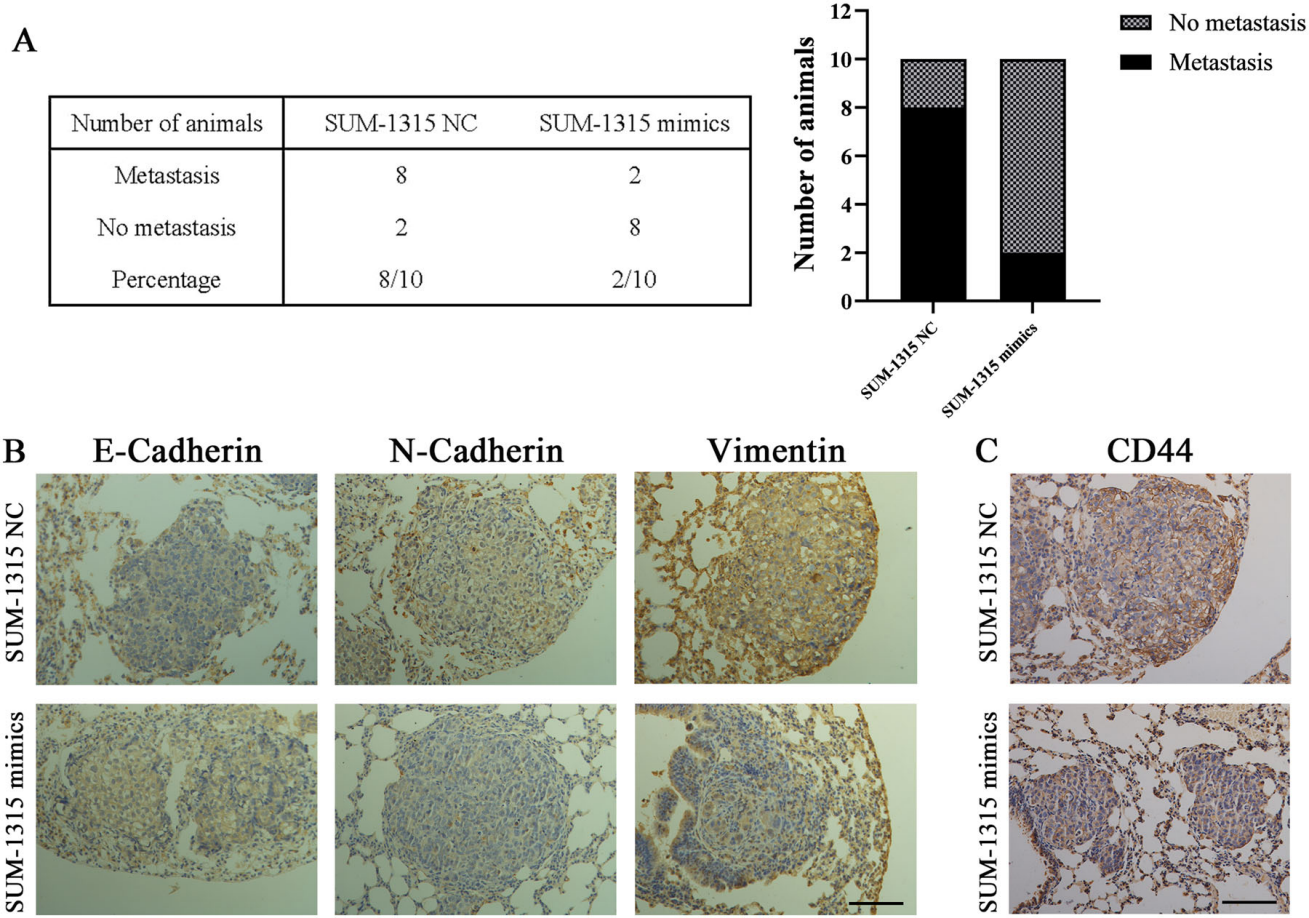
miR-1976 reduced TNBC metastasis in vivo. SUM-1315 transfected with miR-1976 mimics and negative control were injected into the tail vein of female nude mice. **a** Table listed the incidence of metastasis in the nude mice treated with SUM-1315 transfected with miR-1976 mimics and negative control. **b** IHC analyses of E-Cadherin, N-Cadherin, and Vimentin in lung metastases. The positive staining was indicated by a brown color. The expression level of E-Cadherin in the control group was lower than that in the other group injected with miR-1976 mimics, while the expression levels of N-Cadherin and Vimentin in the control group were markedly higher. Scale bars: 100 μm. **c** IHC analyses of CD44 in lung metastases. The positive staining was indicated by a brown color. The expression level of CD44 in the control group was significantly higher than that in the other group injected with miR-1976 mimics. Scale bars: 100 μm.

In order to investigate the effects of miR-1976 in vivo, SUM-1315 transfected with negative control and mimics lentiviruses were injected into the tail vein of female nude mice. After 4 weeks, all mice were euthanized, and lung tissues were removed for further investigation. Metastases were observed in two of ten mice injected with SUM-1315 miR-mimics, while eight of ten mice injected with negative control exhibited metastasis (Fig. 4a). Furthermore, IHC analyses of E-Cadherin, N-Cadherin, and Vimentin in the lung metastases were analyzed. The expression level of E-Cadherin in control group was weaker than that in the other group injected with miR-1976 mimics, while the expression levels of mesenchymal biomarkers (N-Cadherin and Vimentin) in the control group were markedly stronger (Fig. 4b). Similarly, the expression level of CD44 in control group was significantly stronger than those injected with miR-1976 mimics (Fig. 4c). These results indicated that overexpression of miR-1976 might reduce tumor metastasis by inhibiting EMT and CSCs in vivo.

### PIK3CG was a direct downstream target of miR-1976

**Fig. 5:**
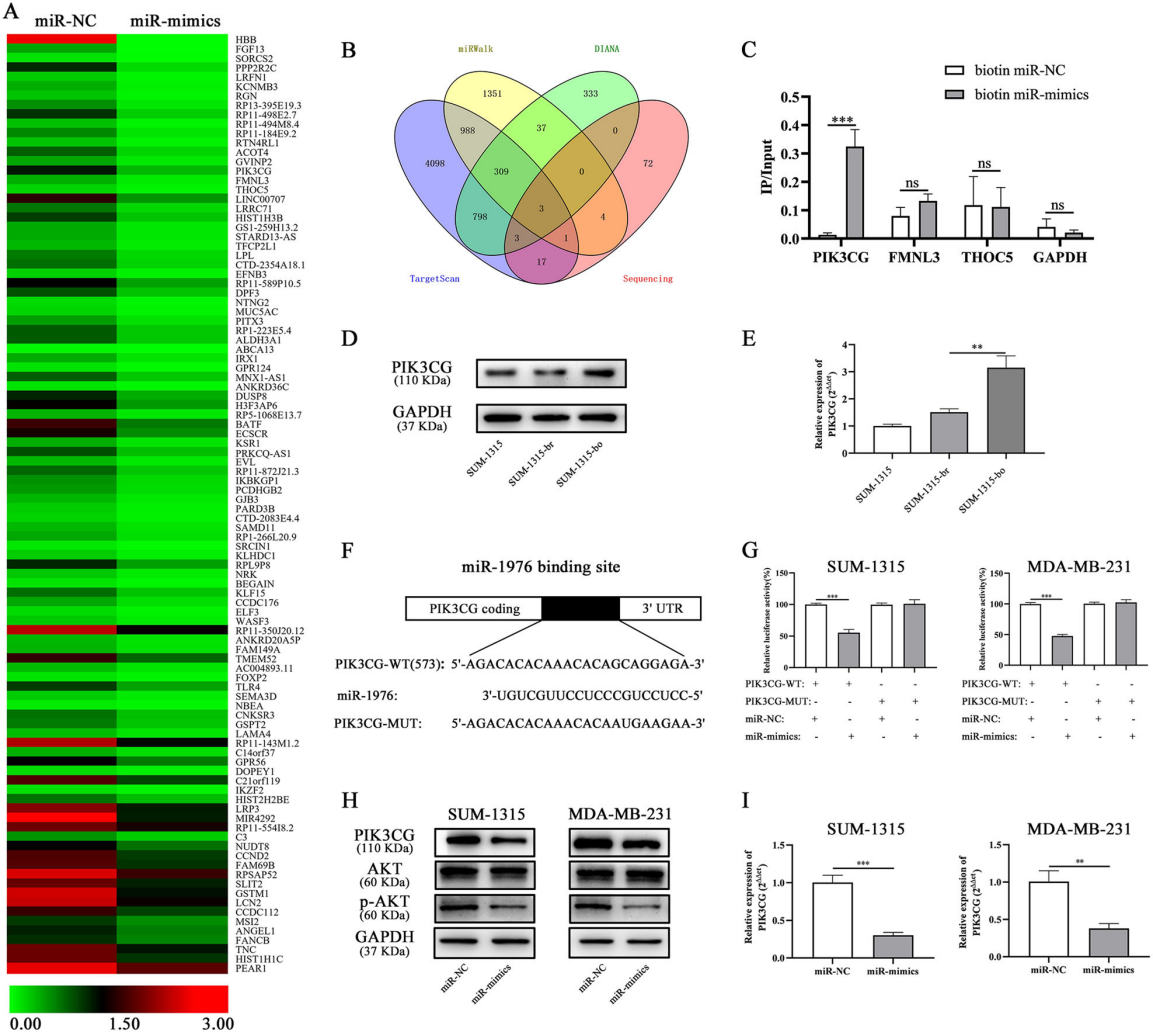
PIK3CG was a direct downstream target of miR-1976. **a** The heatmap showed the transcriptome sequencing of SUM-1315 transfected with miR-1976 mimics and negative control. **b** The Venn-diagram indicates the numbers of genes that overlapped in three publicly available bioinformatics algorithms (TargetScan, miRWalk, and DIANA) and transcriptome sequencing. **c** The 3ʹ-end biotin-labeled miR-1976 mimics and negative control were transfected into SUM-1315 cells, and the mRNA levels of PIK3CG, FMNL3, THOC5, and GAPDH were quantified by qRT-PCR, and the relative immunoprecipitate/input ratios were plotted. **d** The protein expression of PIK3CG in SUM-1315-bo was higher than that in SUM-1315-br. **e** The mRNA expression of PIK3CG in SUM-1315-bo was higher than that in SUM-1315-br. **f** The luciferase reporter constructed that had either a PIK3CG-3ʹ-UTR-WT or PIK3CG-3ʹ-UTR -MUT sequence of the miR-1976 binding site. **g** Luciferase reporter activity revealed that miR-1976 suppressed PIK3CG 3ʹ UTR luciferase activity of wide-type constructs in SUM-1315 and MDA-MB-231 cells. **h** The protein expression levels of PIK3CG, AKT, and p-AKT (a downstream) in SUM-1315 and MDA-MB-231 transfected with miR-1976 mimics were reduced. **i** qRT-PCR analysis showed that miR-1976 mimics decrease the mRNA level of PIK3CG. *p < 0.05, **p < 0.01, ***p < 0.001. The data expressed as the mean ± SD.

To explore the underlying mechanisms of miR-1976, transcriptome sequencing of SUM-1315 transfected with negative control and mimics lentiviruses was performed, and genes with an absolute fold change (FC) > 1.5 and *p*-value ≤ 0.05 were selected (Fig. 5a). Three publicly available bioinformatics algorithms (TargetScan, miRWalk, and DIANA) were used to predict target genes, and 3 genes were overlapped (Fig. 5b). The 3ʹ-end biotinylated negative control and mimics were transfected into SUM-1315 cells. After streptavidin capture, the mRNA levels of PIK3CG, FMNL3, THOC5 and GAPDH were quantified by qRT-PCR, and the relative immunoprecipitate/input ratios were plotted (Fig. 5c). Furthermore, according the mRNA expression profiling analysis of SUM-1315-br and SUM-1315-bo, the expression level of PIK3CG in SUM-1315-bo was higher than that in SUM-1315-br. The protein and mRNA expression of PIK3CG were also verified by western blot and qRT-PCR (Fig. 5d, e).

PIK3CG was identified as a potential target gene of miR-1976, and possible binding site was obtained from database (Fig. 5f). Luciferase reporter assay was constructed that had either a PIK3CG-3ʹ-UTR-WT or PIK3CG-3ʹ-UTR-MUT sequence of the miR-1976-binding site. The relative luciferase activities were decreased after transfected with miR-1976 mimics and PIK3CG-3ʹ-UTR-WT in SUM-1315 and MDA-MB-231 cells, while the luciferase activities were not decreased after transfected with PIK3CG-3ʹ-UTR-MUT (Fig. 5g). The protein expression levels of PIK3CG, AKT and p-AKT (a downstream) were measured in SUM-1315 and MDA-MB-231 transfected with miR-1976 mimics by western blot, and the levels were reduced (Fig. 5h). The mRNA expression level of PIK3CG was measured by qRT-PCR, and the level was reduced after SUM-1315 and MDA-MB-231 transfected with miR-1976 mimics (Fig. 5i). Overall, miR-1976 mimics decreased mRNA and protein expression level of PIK3CG, and it suggested that PIK3CG might be a direct target of miR-1976 and that its expression level was negatively regulated by miR-1976.

### Overexpression of PIK3CG accounted for function of miR-1976

**Fig. 6:**
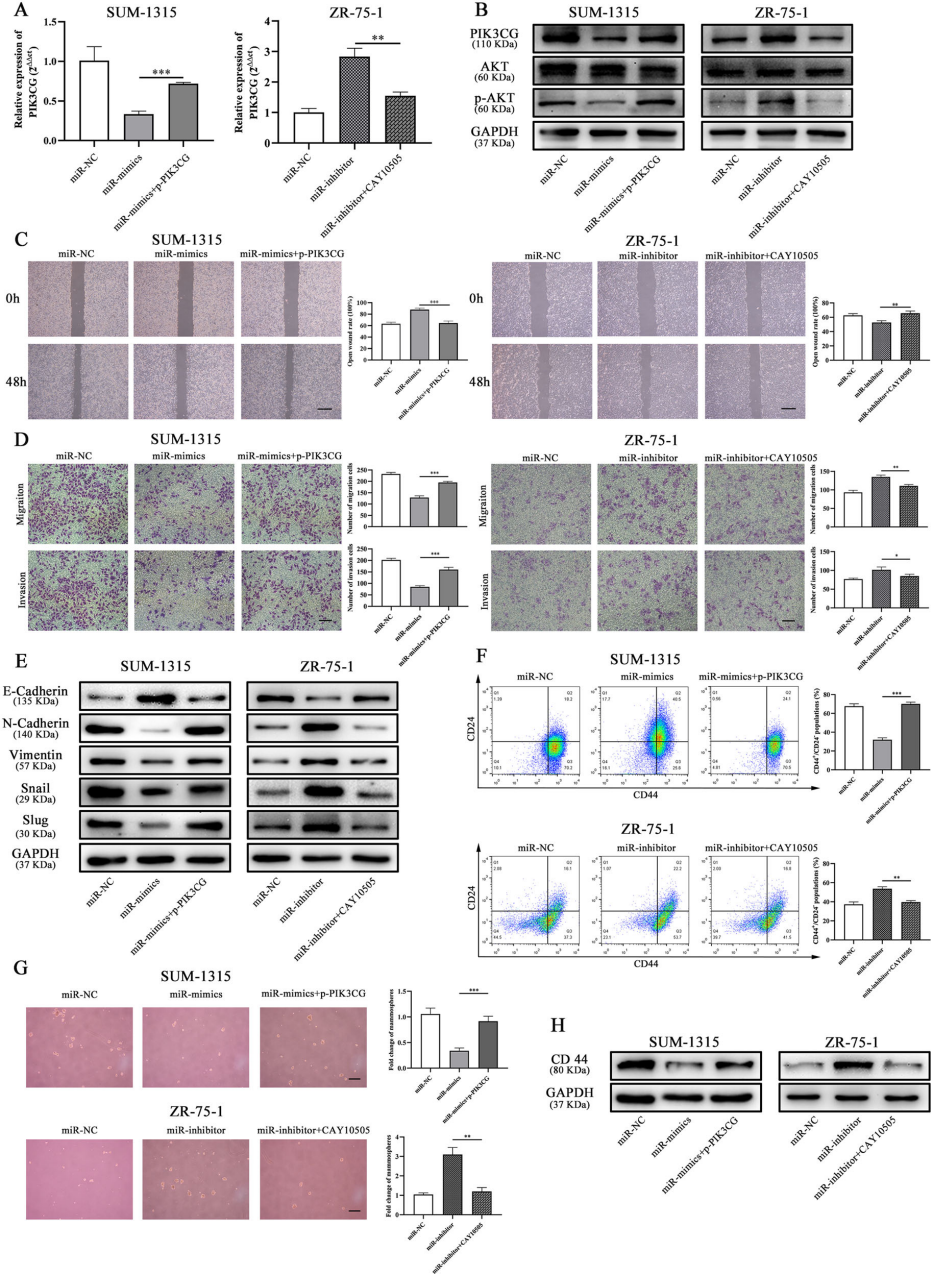
Overexpression of PIK3CG accounted for miR-1976 function in TNBC. **a** The mRNA expression of PIK3CG was measured by qRT-PCR. **b** The protein expression levels of PIK3CG, AKT, and p-AKT were measured by western blot. The properties of EMT in SUM-1315 and ZR-75-1 transfected with miR-NC, miR-mimics, miR-inhibitor, p-PIK3CG, or PIK3CG inhibitor CAY10505 were determined by wound healing assay (**c**), transwell assay (**d**). Scale bars: 100 μm. **e** p-PIK3CG restored the protein expression of the EMT markers and transcription factors, and CAY10505 counteracted the effect of miR-1976 inhibitor. CSC properties of SUM-1315 and ZR-75-1 transfected with miR-NC, miR-mimics, miR-inhibitor, p-PIK3CG, or PIK3CG inhibitor CAY10505 were determined by flow cytometry analysis (**f**) and mammosphere formation assay (**g**). Scale bars: 100 μm. **h** p-PIK3CG restored the protein expression of CD44, and CAY10505 decreased the expression. *p < 0.05, **p < 0.01, ***p < 0.001. The data expressed as the mean ± SD.

To further investigate whether the effects of miR-1976 on EMT and CSCs were mediated by PIK3CG, the expression level of PIK3CG was upregulated by PIK3CG plasmid (p-PIK3CG) and downregulated by PIK3CG inhibitor, CAY10505 (MedChemExpress). As expected, miR-1976 mimics reduced the mRNA and protein level of PIK3CG in SUM-1315 cells, and this effect was reversed by p-PIK3CG, while miR-1976 inhibitor increased the mRNA and protein level of PIK3CG in ZR-75-1 cells, and this effect was reversed by CAY10505 (Fig. 6a, b). The wound healing assay and transwell assay were performed, and we found that p-PIK3CG could attenuated the inhibitory effect of miR-1976 in EMT, and CAY10505 could suppress the effects (Fig. 6c, d). The expression levels of the epithelial marker, mesenchymal markers, and transcription factors in SUM-1315 affected by miR-1976 mimics were reversed by p-PIK3CG. In addition, CAY10505, PIK3CG inhibitor, could reverse the effects of miR-1976 inhibitor in ZR-75-1 cells (Fig. 6e). The effects of PIK3CG on proliferation and cell death were also analyzed. p-PIK3CG promoted cell proliferation and reduced cell apoptosis, and CAY10505 suppressed cell proliferation and induced cell apoptosis (Supplementary Fig. S4).

Similarly, p-PIK3CG could markedly increase the populations of CD44^+^/CD24^−^ and the number of mammospheres in SUM-1315 cells transfected with miR-1976 mimics. In ZR-75-1 cells transfected with miR-1976 inhibitor, CAY10505 could partially reduce the populations and the number of mammospheres (Fig. 6f, g). Consistently, p-PIK3CG upregulated the protein expression of CD44 in SUM-1315 cells transfected with miR-1976 mimics, and CAY10505 reduced the expression level in ZR-75-1 cells transfected with miR-1976 inhibitor (Fig. 6h). Taken together, it suggested that miR-1976 exerted its antitumor effects in TNBC by targeting PIK3CG, and PIK3CG could be a functional target of it.

## Discussion

TNBC is defined as the subtype lacking the expression of ER, PR, and HER2^14^, and lacks effective targeted therapy, so the prognosis of TNBC is worse than other subtypes. Metastasis is the primary cause of death in TNBC patients, and a great challenge in clinical treatment^1^. In 1889, Stephen Paget proposed the “seed and soil” hypothesis, and thought that tumor metastasis was the result of the development of metastatic tumor cells (seeds) in the target organs (soil)^15,16^. The interactions between tumor cells and target organs play an important role in metastasis, that is, metastasis is a two-sided function. We established a novel mouse model containing normal human breast and bone tissues^12^. This model simulated the microenvironment of TNBC metastasis in human, including the microenvironment of local tumor and target organs. We compared the different miRNA expression between SUM-1315-br and SUM-1315-bo, and miR-1976 was selected and verified in clinical tissues and databases.

Previous studies have described the role of miR-1976 in non-small cell lung cancer, functioning as a tumor suppressor and serving as a prognostic indicator^17^. However, very little was known about the biological effects on TNBC. We explored it and found that miR-1976 knockdown significantly increased the wound healing rate and cell migration, promoted proliferation, reduced cell apoptosis, and enhanced the populations of CD44^+^/CD24^−^ in vitro. Moreover, miR-1976 inhibitors downregulated the epithelial marker, E-Cadherin, and upregulated mesenchymal markers and transcription factors, N-Cadherin, Vimentin, Snail and Slug. The effects of miR-1976 on proliferation and cell death could contribute to the results in EMT and CSCs. These effects were also verified in vivo. We adopted two different experimental animal models to study the roles of miR-1976 in tumor metastasis. The metastatic model was used to simulate human microenvironment and screen different indicators, while the tail vein injection model was a general model in researching tumor metastasis^18,19^. Immunohistochemical analyses of EMT markers and CD44 in the lung metastases were analyzed. The expression levels of E-Cadherin in the control group were significantly lower than those injected with miR-1976 mimics, while the expression levels of N-Cadherin, Vimentin, and CD44 were markedly higher. Taken together, these results indicated that miR-1976 acted as a suppressor miRNA, exerting an important effect on TNBC progression.

MiR-1976 knockdown markedly promoted migration and invasion, acquiring aggressive traits and strengthening mesenchymal properties. Mesenchymal cells infiltrate into distant organs and then transit into epithelial cells to form macroscopic metastatic foci. EMT enables the steps of metastasis, including local invasion and dissemination to target organs^20,21^. Several studies have shown that EMT could accelerate progression by the increase of proliferation, suppression of apoptosis, induction of cancer stem cells, augmented angiogenesis, and immunosuppression^22,23^. Metastasis on target organs is thought to be associated with self-renewal^24,25^. CSCs are a small group of tumor cells with self-renewal and differentiation capability similar as normal stem cells, reproducing the heterogeneity of original tumors^26–28^. Accumulating evidences have revealed that CSCs have long-term proliferative potential and are important mediators of tumor metastasis and cancer relapse^29^. CD44 and CD24 are the most common CSC markers in breast cancer^30^, and CD44^+^ and CD24^–/low^ are associated with anti-cancer drug resistance, tumor recurrence, and metastasis. In our study, we found miR-1976 knockdown markedly promoted EMT and CSC properties.

PIK3CG was screened as a target gene of miR-1976 by biotinylated miRNA capture, and verified by luciferase reporter assay. Overexpression of PIK3CG counteracted the inhibition effect of miR-1976 mimics on EMT and CSC properties, and PIK3CG inhibitor CAY10505 could reverse the effects of miR-1976 inhibitor. MiR-1976 regulated the expression level of PIK3CG via binding to the 3ʹUTR of the mRNAs resulting in post-transcriptional repression or degradation. PIK3CG is considered as a cancer-promoting gene in a variety of tumors, and it has been reported that PIK3CG promoted metastasis and invasion^31,32^. At present, the potential targets of TNBC are mainly abnormal signal transduction pathways or overexpressed proteins, such as mTOR (mammalian target of rapamycin) inhibitors everolimus^33^, which has been used in clinical trials. MTOR is an important protein kinase and is a downstream of PI3K/Akt signaling pathway that regulates the proliferation, invasion and metastasis by activating ribosomal kinases. Our results suggested that PIK3CG inhibitor may also be a treatment target for TNBC metastasis, achieving similar effects to mTOR inhibitor.

In conclusion, we have identified that miR-1976 was downregulated in TNBC. MiR-1976 knockdown induced TNBC metastasis by promoting EMT and CSCs. These effects of miR-1976 were dependent on its direct regulation of PIK3CG. These findings may reveal mechanisms of TNBC metastasis, and represent a potential treatment target for patients with TNBC.

## Supplementary information

Supplementary figure legends

Fig. S1

Fig. S2

Fig. S3

Fig. S4

Table. S1
