# Supplementary material for: MiR-1976 knockdown promotes epithelial–mesenchymal transition and cancer stem cell properties inducing triple-negative breast cancer metastasis
Source: Cell Death Dis. 2020 Jul 3;11(7):500. doi: 10.1038/s41419-020-2711-x (PMC7335055; doi:10.1038/s41419-020-2711-x)
Supplement: Supplementary file 1 — Supplementary figure legends [file 41419_2020_2711_MOESM1_ESM.docx]

**Supplementary figure legends**

**Fig. S1 miR-1976 was down-regulated in SUM-1315-bo and TNBC cell lines. a.** The expression levels of miR-1976 in SUM-1315, SUM-1315-br (derived from orthotopic breast tumor), and SUM-1315-bo (derived from metastatic bone tumor). **b.** The expression levels of miR-1976 in TNBC cell lines and hormone receptor positive cell lines. ****p* < 0.001. The data expressed as the mean ± SD.

**Fig. S2 MiR-1976 knockdown promoted proliferation and decreased cell death in vitro.** Cell proliferation was determined by cell viability assay in SUM-1315 and MDA-MB-231 transfected with miR-mimics, and in ZR-75-1 and MCF-7 transfected with miR-inhibitors (**a.**). The Ki-67 index decreased in SUM-1315 and MDA-MB-231 transfected with miR-mimics, and increased in ZR-75-1 and MCF-7 transfected with miR-inhibitors (**b.**). Flow cytometry analysis of the effect of miR-1976 expression alteration on cell apoptosis (**c.**). **p* < 0.05, ***p*<0.01, ****p* < 0.001. The data expressed as the mean ± SD.

**Fig. S3 Negative controls for CD44 and CD24 in flow cytometry analysis.** Cells stained with the APC and PE isotype control antibodies were used as negative controls.

**Fig. S4 Inhibition of PIK3CG expression accounted for the effects of miR-1976 in proliferation and cell death.** Cell proliferation of SUM-1315 and ZR-75-1 transfected with miR-NC, miR-mimics, miR-inhibitor, p-PIK3CG, or PIK3CG inhibitor CAY10505 was determined by cell viability assay (**a.**) and flow cytometry analysis of Ki-67 (**b.**). **c.** The cell apoptosis of SUM-1315 and ZR-75-1 after co-transfection was measured in flow cytometry analysis. **p* < 0.05, ***p*<0.01, ****p* < 0.001. The data expressed as the mean ± SD.
