# Supplementary figures and images for: MiR-1976 knockdown promotes epithelial–mesenchymal transition and cancer stem cell properties inducing triple-negative breast cancer metastasis
Source: Cell Death Dis. 2020 Jul 3;11(7):500. doi: 10.1038/s41419-020-2711-x (PMC7335055; doi:10.1038/s41419-020-2711-x)

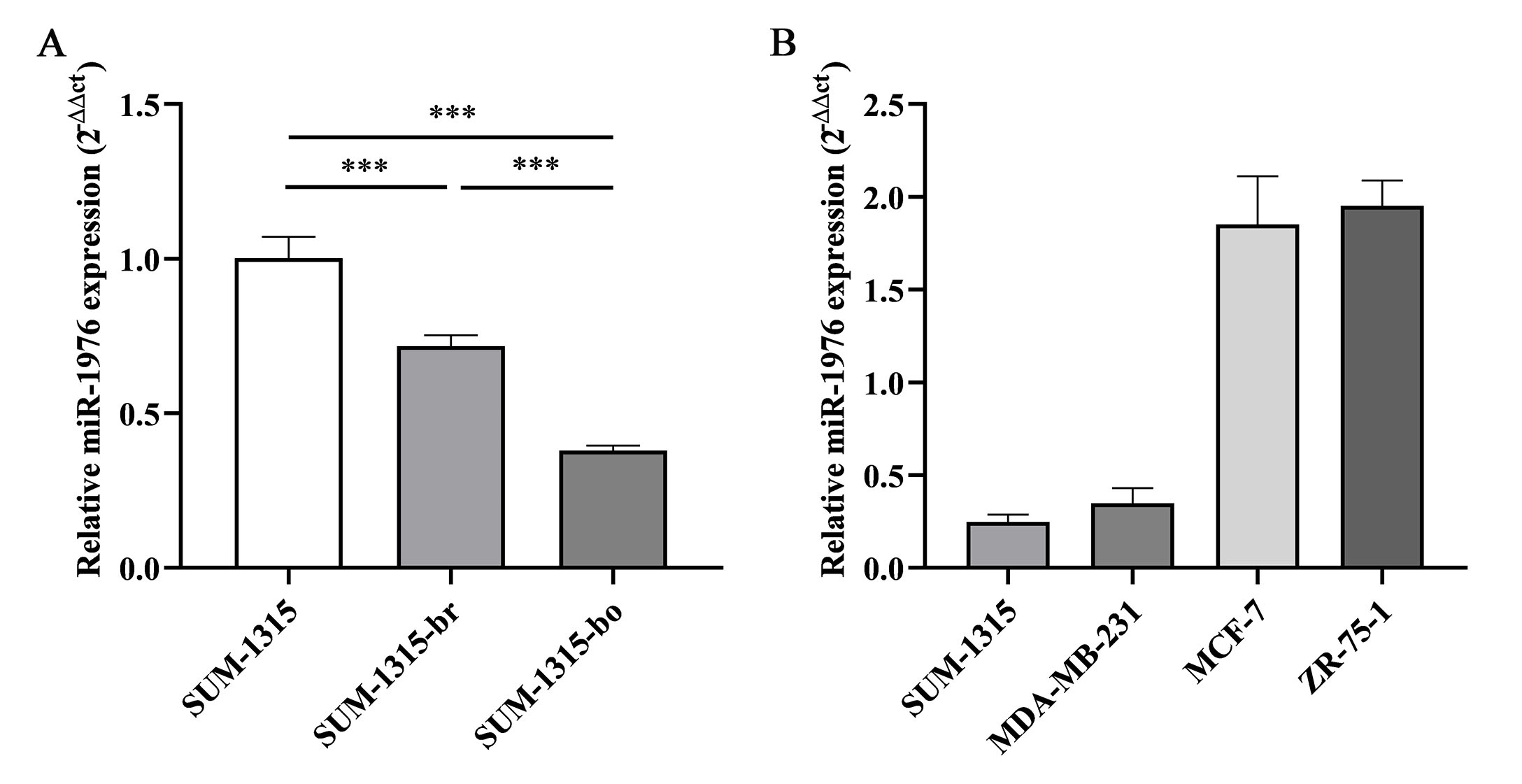

Supplement: Supplementary file 2 — Fig. S1 [file 41419_2020_2711_MOESM2_ESM.tif]

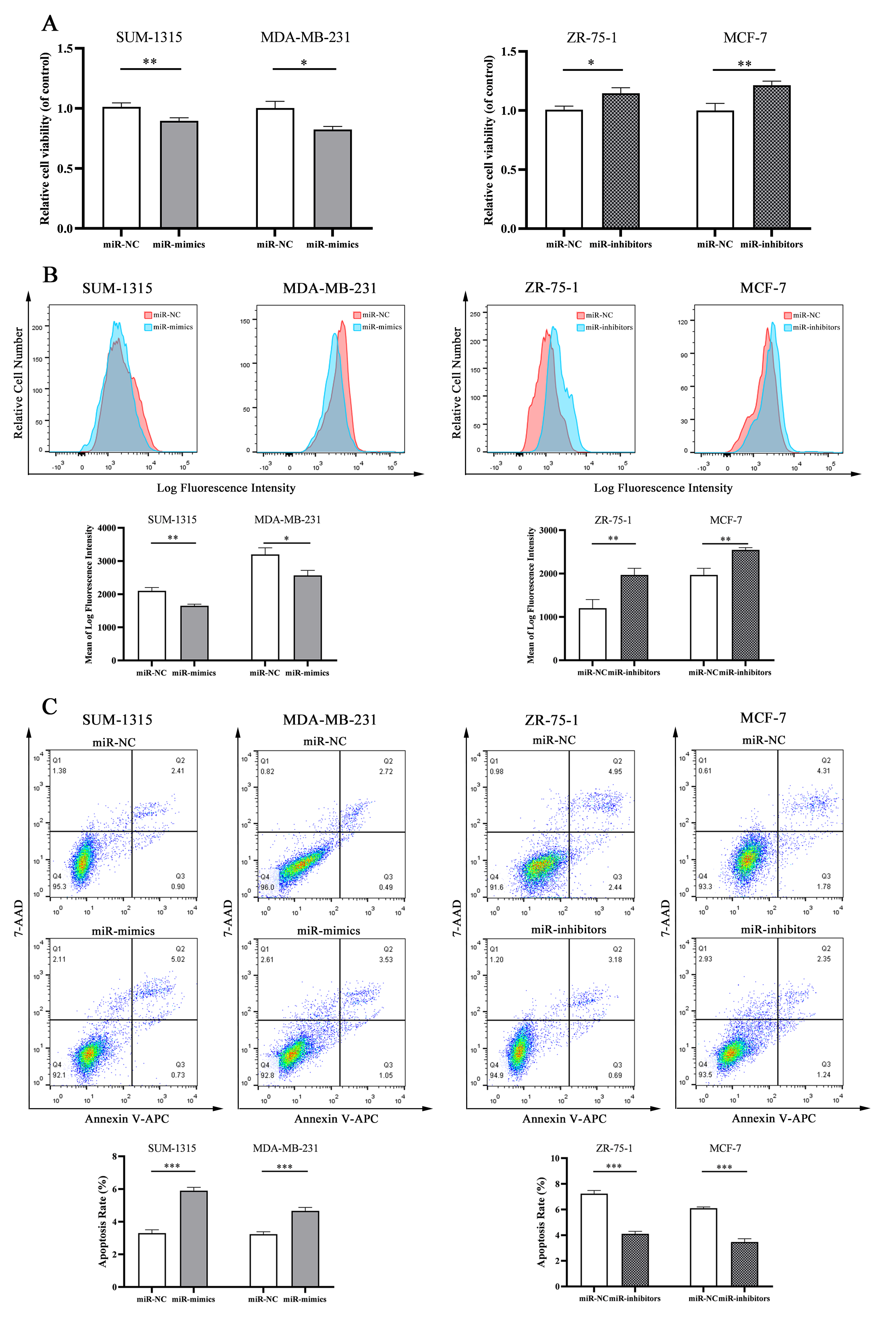

Supplement: Supplementary file 3 — Fig. S2 [file 41419_2020_2711_MOESM3_ESM.tif]

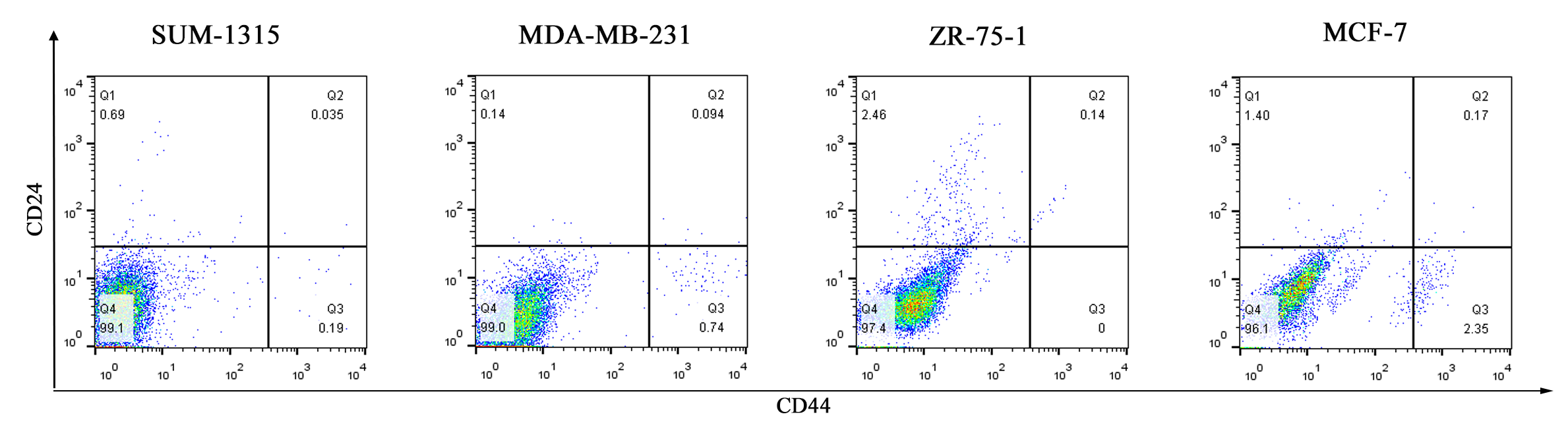

Supplement: Supplementary file 4 — Fig. S3 [file 41419_2020_2711_MOESM4_ESM.tif]

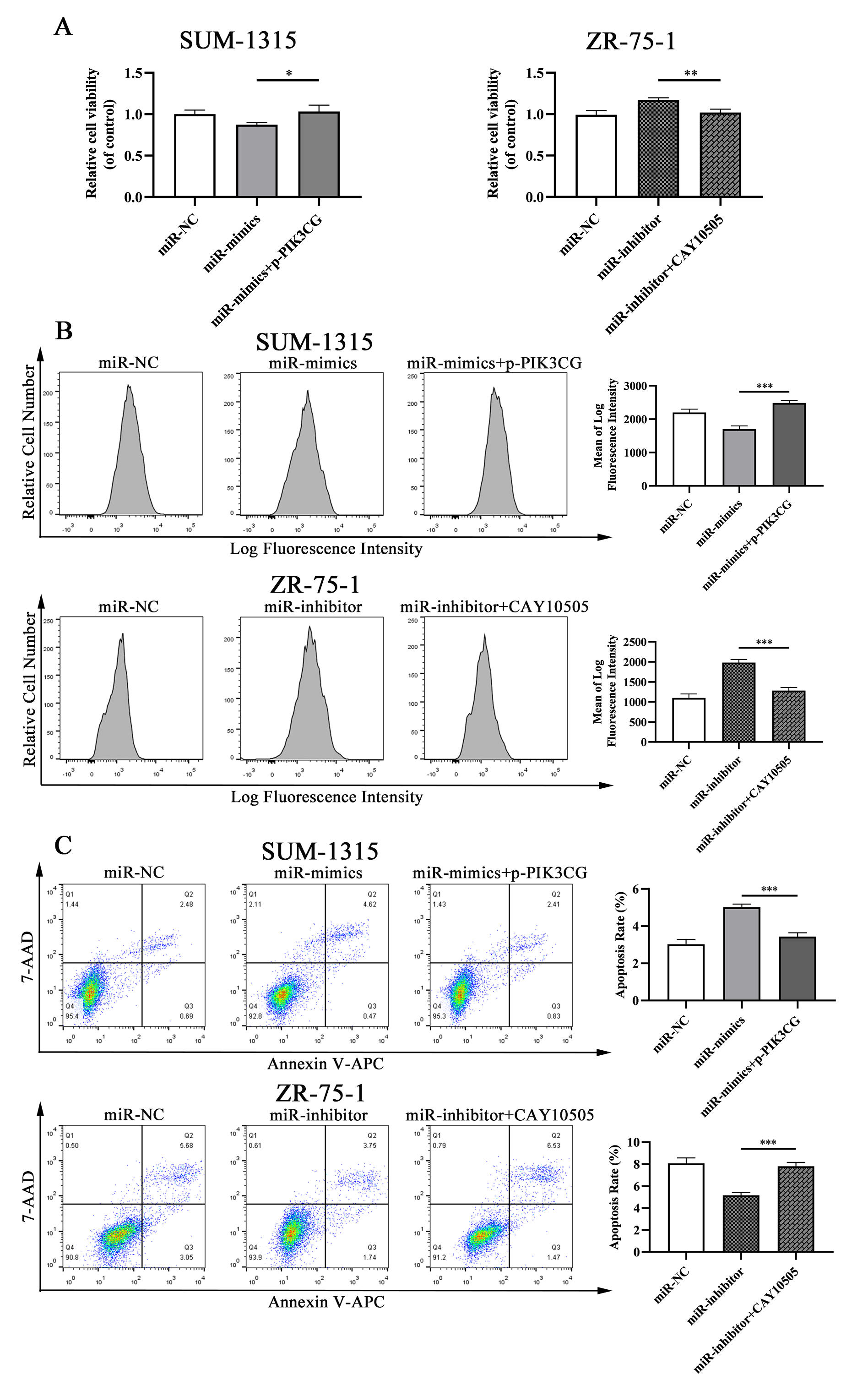

Supplement: Supplementary file 5 — Fig. S4 [file 41419_2020_2711_MOESM5_ESM.tif]
