# Supplementary material for: MiR-1976 knockdown promotes epithelial–mesenchymal transition and cancer stem cell properties inducing triple-negative breast cancer metastasis
Source: Cell Death Dis. 2020 Jul 3;11(7):500. doi: 10.1038/s41419-020-2711-x (PMC7335055; doi:10.1038/s41419-020-2711-x)
Supplement: Supplementary file 6 — Table. S1 [file 41419_2020_2711_MOESM6_ESM.docx]

**Table. S1** Clinical characteristics of individuals contributing TNBC tissues and adjacent normal tissues in the study.

| Characteristics | Group | Number |
| --- | --- | --- |
| Gender | Female | 35 |
|  | Male | 0 |
| Age | ≤50 | 15 |
|  | >50 | 20 |
| Histological grade | I-II | 16 |
|  | III | 19 |
| Subtypes | TNBC | 35 |
|  | Luminal | 0 |
|  | HER2 | 0 |
| Lymph nodes | ≤1 | 23 |
|  | >1 | 12 |
| Ki-67 | ≤20% | 8 |
|  | >20% | 27 |
